# Prevalence of familial cluster headache: a systematic review and meta-analysis

**DOI:** 10.1186/s10194-020-01101-w

**Published:** 2020-04-25

**Authors:** Emer O’Connor, Benjamin S. Simpson, Henry Houlden, Jana Vandrovcova, Manjit Matharu

**Affiliations:** 1 Department of Neuromuscular Disorders, UCL Institute of Neurology, DMN, Institute of Neurology, Queen Square, London, WC1N 3BG UK; 2 Headache and Facial Pain Group, UCL Institute of Neurology, Queen Square and The National Hospital for Neurology and Neurosurgery, Queen Square, London, UK; 3 UCL Division of Surgery and Interventional Science, University College London, London, UK

**Keywords:** Cluster headache, Trigeminal autonomic cephalalgia, Headache, Genetics, Gene, Systematic review, Meta-analysis, Family history, Heritability, Heredity, Inheritance

## Abstract

**Introduction:**

The population rate of familial cluster headache (CH) has been reported to be as high as 20% however this varies considerably across studies. To obtain a true estimate of family history in CH, we conducted a systematic review and meta-analysis of previously published data.

**Methods:**

Our systematic review involved a search of electronic databases (Medline, EMBASE, PubMed, CINAHL) to identify and appraise studies of interest utilising the PRISMA (Preferred Reporting Items for Systematic Review and Meta-Analysis) guidelines. To further ameliorate the accuracy of our analysis we included an additional unpublished cohort of CH patients recruited at a tertiary referral centre for headache, who underwent detailed family history with diagnostic verification in relatives. Data was extracted and meta-analysis conducted to provide a true estimation of family history.

**Results:**

In total, we identified 7 studies which fulfilled our inclusion criteria. The estimated true prevalence of CH patients with a positive family history was 6.27% (95% CI:4.65–8.40%) with an overall I^2^ of 73%. Fitted models for gender subgroups showed higher estimates 9.26% (95% CI: 6.29–13.43%) in females. However the I^2^ for the female model was 58.42% and significant (*p* = 0.047).

**Conclusion:**

Our findings estimate a rate of family history in CH to be approximately 6.27% (95% CI: 4.65–8.40%). While estimates were larger for female probands, we demonstrated high heterogeneity in this subgroup. These findings further support a genetic role in the aetiology of CH.

## Background

Often referred to as the “suicide headache”, cluster headache (CH) has been described as one of the most painful conditions a human can experience, with female sufferers reporting pain more severe than childbirth [1]. Typically, it presents with severe strictly unilateral pain in the distribution of the first branch of the trigeminal nerve. The pain characteristically lasts from 15 to 180 min with associated observable cranial autonomic features [2]. Considerably rare, it has an estimated prevalence of approximately 1 in 1000, however its occurrence varies across geographical regions and has been reported to be as high as 1 in 500 [3–8]. Those with a family history of CH appear to have an increased risk of developing the condition [9–12] . Estimations of the presence of a positive family history amongst sufferers varies across studies. For example, in one cohort the familial prevalence was 2.3%, with a low Falconer’s heritability index, indicating a high likelihood of an environmental cause [13]. Others, however, estimate a positive family history in up to 20% of patients, inferring a 39 fold relative risk [14]. Inter-familial clinical variability has also been observed, with an earlier age of onset reported in the offspring of parents with CH, inferring the possibility of anticipation [15]. There also appears to be a higher proportion of female sufferers in familial cases [16]. These findings have provided a basis for familial studies and genetic association studies in genes with a putative role in the pathophysiology of CH [17–24]. The purpose of this study was to perform a systematic appraisal and meta-analysis of all studies in addition to presenting original data reporting a prevalence of familial CH.

## Methods

### Systematic review

This systematic review was registered with PROSPERO, the International Prospective Register of Systematic Reviews (registration number CRD42019157309) and carried out in accordance with the guidelines for Preferred Reporting Items for Systematic Reviews and Meta-analysis Protocols (PRISMA- P) [25]. All published studies of interest were identified through a search involving the following electronic databases: MEDLINE, PUBMED, EMBASE, CINAHL.

A pre-defined search strategy was formulated which included a combination of relevant medical subject headings (MeSH), associated synonyms and free text [26]. To identify studies reporting a family history the following terms were used; “family” OR “familial” OR “hereditary” OR “heritability” OR “hereditability” OR “inherit” OR “inherited” OR “genetic” OR “genes” OR “gene”. These were added to terms for CH including “Trigeminal Autonomic Cephalalgia” OR “TACS” OR “Cluster Headache” OR “cluster headaches” combined using the ‘AND’ operator. To ensure a robust review, references from cited articles were also screened. Finally, experts were also consulted to identify additional missed literature. The details of the search strategy used for individual databases is summarised in Table 1.

**Table 1:** Search criteria used for databases to identify articles on family history in CH

DATABASE	SEARCH TERM	Results
1. Pubmed	*(“trigeminal autonomic cephalalgias”[MeSH] OR “Trigeminal Autonomic Cephalalgia” OR “Trigeminal Autonomic Cephalalgias” OR “TACS” OR “Cluster Headache”[MeSH] OR “Cluster Headache” OR “cluster headaches”) AND (“family”[MeSH] OR “family” OR “familial” OR “hereditary” OR “heritability” OR “hereditability” OR “inherit” OR “inherited” OR “genetic” OR “genes”[MeSH] OR “genes” OR “gene”)*	408
2. Medline	(exp trigeminal autonomic cephalalgia/ or exp cluster headache/) OR (TACS or Cluster Headache*).mp AND (family or familial).mp. OR (hered* or heritability or inherit* or genetic or genes or gene).mp.	391
3. EMBASE	(Trigeminal autonomic cephalalgia/ or exp cluster headache/)OR (trigeminal autonomic cephalalgia* or TACS or cluster headache*)mp AND (family or familial or hered* or heritability or inherit or gene or genes or genetic*).mp.	946
4. CINAHL	(MH “Trigeminal Autonomic Cephalalgias+”) OR (trigeminal autonomic cephalalgia* OR TACS OR cluster headache*) AND ((family or familial) OR (heredit* or heritability) OR inherit* OR (gene or genes or genetic*))	283

### Eligibility criteria and data extraction

All studies reporting the prevalence of familial CH within a defined cohort of CH patients were included in the analysis. The inclusion criteria defined a positive family history as a clinical diagnosis of CH, in one or more affected individuals, who were a first or second-degree relative. To avoid an over representation of familial history, only studies that confirmed a diagnosis of CH in an affected relative were included in the systematic review. All abstracts were independently analysed by two authors and those fulfilling the eligibility criteria were included for full-text review. A separate assessment of the included studies was conducted by two authors independently and the following data was extracted for analysis: study design, year of publication, population studied, methodology of data acquisition, diagnostic criteria employed, number of participants, gender ratio, percentage reporting a family history and gender ratio of patients with familial CH (Table 2).

**Table 2:** Data extracted from identified studies

Country	Authors	Study Design	Diagnostic Criteria	Method of data acquisition	Sample size (n)	Probands with FAMILY HISTORY of CH (n, %)	Probands with FAMILY HISTORY (M)	Probands with FAMILY HISTORY (F)
**USA**	Kudrow and Kudrow (1994) [27]	Retrospective Study	• Adhoc Committee on classification of headache 1962 • ICHD-1	• Proband interview • Semi-structured phone interview • Direct examination	300	26 (8.6%)	7	19
**Denmark**	Russell et al (1996) [28]	Retrospective Study	• ICHD-1	• Questionnaire • Semi-structured phone interview • Direct examination	366	25 (6.8%)	17	9
**Italian**	Montagna et al (1998) [13]	Retrospective Study	• ICHD-1	• Semi-structured phone interview	222	5 (2.25%)	NA	NA
**French**	El Amrani et al (2002) [29]	Consecutive case- series	• ICHD-1	• Direct examination	220	44 (10.75%)	12	8
**Italian**	Torelli and Manzoni (2003) [30]	Retrospective Study	• ICHD-1	• Semi-structured phone interview	186	20 (4.34%)	30	8
**Italian**	Taga et al (2015) [31]	Retrospective Study	• ICHD3β	• Clinical documentation	691	40 (4.92%)	28	12
**Italian**	Leone et al (2001) [14]	Retrospective Study	• ICHD-1	• Semi-structured phone interview Direct examination	785	44 (20%)	29	15
**UK**	O’Connor et al (2020)^a^	Retrospective Study	• ICHD3β	• Semi-structured phone interview • Direct examination	645	48 (7.44%)	35	13

*CH* cluster headache, *F* Female, *ICHD* International Classification of Headache Disorders, *M* Male, *NA* Not available

^a^O’Connor et al. represents unpublished local cohort

To exclude the risk of bias, all eligible studies were independently analysed using a modified Newcastle – Ottawa appraisal checklist, a tool designed to appraise cohort studies on three main areas: the selection of the study groups, the comparability of these groups; and the ascertainment outcome for [32]. The total score of the modified version is limited to 7 stars with removal of sections pertaining only to longitudinal studies (Table 3). All seven studies scored 6 or higher in our risk of bias assessment, demonstrating a low risk of bias, therefore all seven studies were included for meta-analysis.

**Table 3:** Modified Newcastle-Ottawa Quality Assessment Scale for cohort studies with awarded stars per category

Study	Selection	Comparability	Outcome	Total number of stars
Kudrow and Kudrow (1994) [27]	★★★★	★★	★	7
Russell et al. (1996) [28]	★★★★	★	★	6
Montagna et al. (1998) [13]	★★★★	★	★	6
El Amrani et al. (2002) [29]	★★★★	★	★	6
Torelli and Manzoni (2003) [30]	★★★★	★★	★	7
Taga et al. (2015) [31]	★★★★	★	★	6
Leone et al. (2001) [14]	★★★★	★	★	6

A maximum of 7 stars can be awarded in total. Selection category = maximum of 4 stars. Comparability = maximum of 2 stars. Outcome = maximum of 1 star

### Unpublished cohort

We included an additional unpublished cohort of patients who attended the headache clinic at the National Hospital for Neurology and Neurosurgery (Queen Square, London, UK) between January 2007 and April 2017. All consecutive patients diagnosed with CH, in accordance with ICHD3β and met our inclusion criteria were recruited with informed consent and underwent a detailed family history as part of their clinical assessment. A diagnosis of CH was confirmed in family members either in clinic or using a semi-structured phone interview based on the ICHD3β criteria. In cases where relatives were uncontactable or deceased, only those with a diagnosis of CH confirmed by a neurologist were deemed eligible.. A total of 645 patients were included in the study. Of these, 456 (70.69%) were male. A family history of CH was reported in 66 patients (10.2%).18 cases were excluded as relatives did not fulfil the ICHD3β criteria for CH or were uncontactable. Overall, 48 (7.44%) individuals had a confirmed family history of CH.

### Statistical analysis

#### Estimation of relative proportion of effected probands with positive family history of CH

Of the seven identified studies, we extracted the total number of affected probands with a first or second degree relative with a clinical diagnosis of CH and the total number of cases in the study [13, 28–31, 33]. The raw/direct proportions were calculated and the distribution of untransformed, logit and double-arcsine transformed proportions were compared. The distributions of the proportions were assessed for normality using density plots and tested using the Shapiro-Wilk test. Logit-transformed proportions most resembled a normal distribution therefore, this transformation was used for the analysis. Due to high inter-study variation and high I^2^, a random-effects model was fitted for estimation of family history in CH. After fitting a model to all relevant studies, leave-one-out analyses (LOO) and accompanying diagnostic plots were used to identify influential studies including: externally studentized residuals, difference in fits values (DFFITS), Cook’s distances, covariance ratios, LOO estimates of the amount of heterogeneity, LOO values of the test statistics for heterogeneity, hat values and weights. Briefly, each study was removed one at a time, and the summary proportion is re-estimated based on the remaining n-1 studies. Studies with a statistically significant influence on the fitted model were removed as outliers and the model was re-fitted. All data analysis and visualisation was performed using the R statistical environment (version 3.6.1, 2019-07-05) using the “metafor” and “meta” packages. The analysis was performed as outlined by Wang [34].

We performed a gender-segregated analysis that included all studies from our initial analysis which also reported the prevalence for males and females separately. In total, studies had gender segregated numbers: *Kudrow and Kudrow (1994), Russell (1996), Leone (2001), El Amrani (2002) Taga (2015)* and our unpublished cohort: *O’Connor (2020*). We represented each study with a male and female estimate of family history prevalence. *Leone (2001)* was identified as an outlier in our initial analysis. We continued to exclude this study for two reasons: our method of analysis results in two separate entries per study (one male, one female), causing influential studies to be over-represented which may skew outlier analysis, and secondly, these estimates are not truly independent. Based on the identified literature, we chose not to assume a common between-study variance component across males and females, therefore, we did not pool within-group estimates of τ^2^. Additionally, there were five studies per subgroup, allowing a moderately stable estimate of τ^2^ within each subgroup. We, therefore, used a mixed-effects model whereby, all summary effect sizes where calculated using separate τ^2^ within each subgroup (males and females), then two separate random effects models were fitted. We then combined the estimated statistics from each model and fitted a fixed-effect model as outlined by Wang [34].

## Results

### Systematic review

**Fig. 1:**
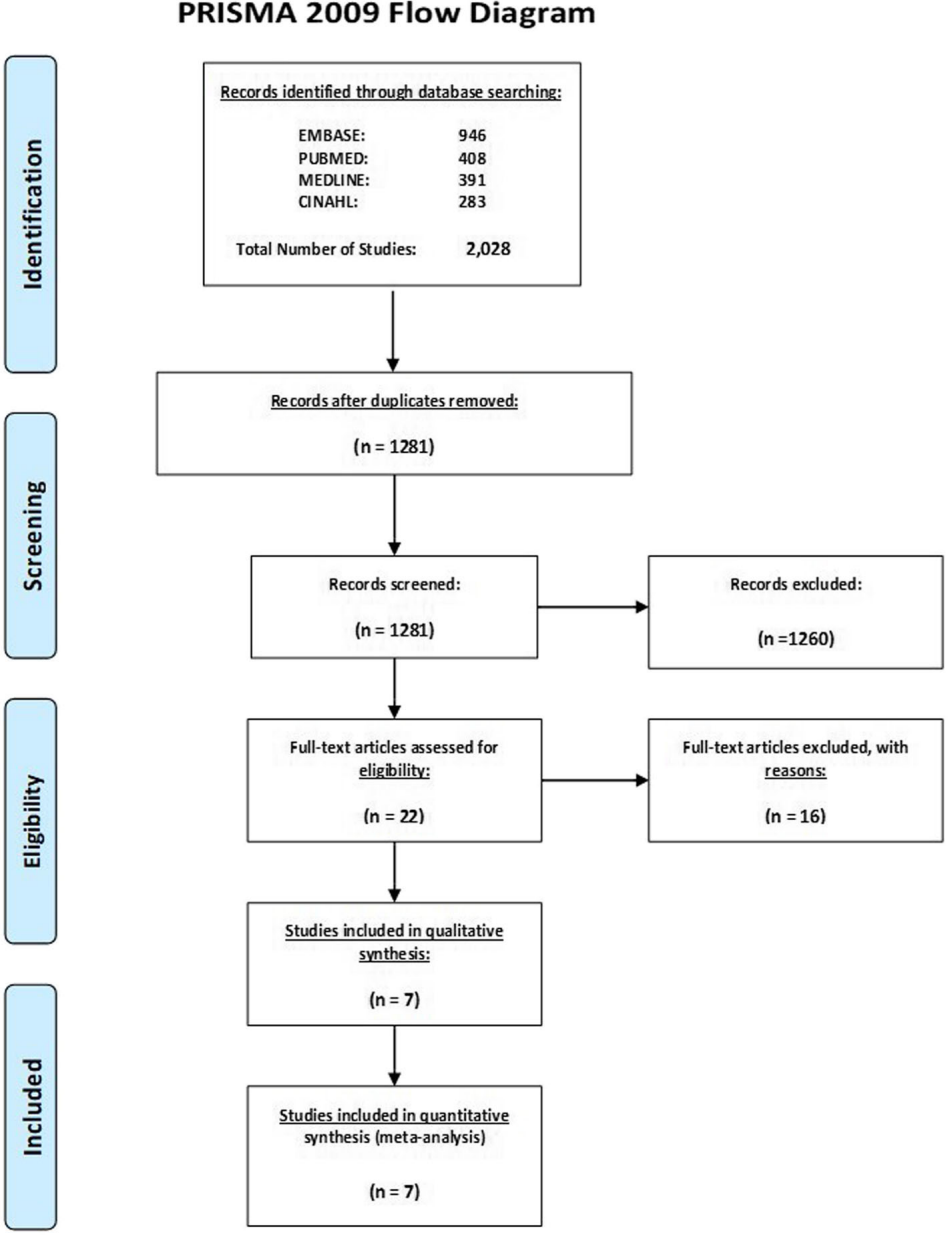
Preferred Reporting Items for Systematic Reviews and Meta-Analyses: The PRISMA Statement. Schematic showing breakdown of screening process. 10.1371/journal.pmed1000097

Following the removal of duplicates, the search strategy identified 1281 studies, all of which were published between 1994 and 2015 (Fig. 1). Following a screening process which excluded 1260 unsuitable abstracts, 22 full-text articles were assessed for eligibility and 7 were selected for inclusion. To avoid over estimating family history, 15 of the 22 studies were removed due to inadequate clinical confirmation in affected relatives. The remaining full texts consisted of 7 cohort studies with an estimated prevalence of family history of CH ranging from 4.9% to 26.3%. After being supplementation with local cohort data [O′ Connor (2020)], the included studies consisted of a total of 3415 CH patients, 238 of which reported a positive family history of CH. Table 2 summarises the extracted data.

### Estimation of relative proportion of effected probands with positive family history of CH

**Fig. 2:**
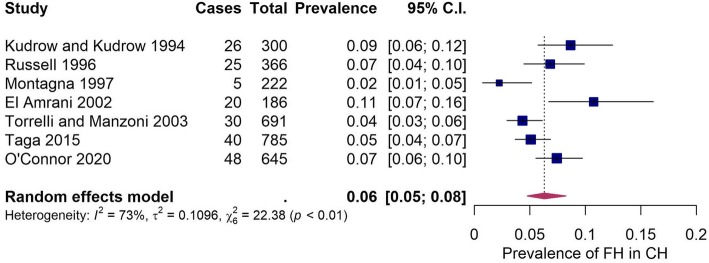
A random effects model was fitted which estimates the true prevalence of family history in cluster headache patients. The study author and year (study), total number of cases with a positive family history (cases), total number of participants (total), prevalence proportion (prevalence) and 95% confidence intervals (95% C.I) are displayed along with measures of study heterogeneity. All values rounded to one significant figure

In order to estimate the true prevalence of family history in patients with CH, we selected studies that had quantified the number of first and/or second degree relatives suffering from CH and had also confirmed these clinical diagnoses. The study data was transformed using the logit-transformation and normality was confirmed using density plot (supplementary Figure 1) and Shapiro-Wilk test (*p* = 0.9889). A random-effects model was fitted and identified a high degree of study heterogeneity (I^2^ = 90.95%, *p* < 0.01). The random effects model which included all the identified studies estimated the prevalence of family history in CH patients to be 7.21% (95% CI:4.69–10.92%) (supplementary Figure 2). Inspection of the externally studentized residuals indicated that the *Leone* et al *(2001)* study had a high z-value (3.05) and therefore may be an outlier. Diagnostic plots also indicated the presence of an outlier and are shown in supplementary Figure 3. Leave one out (LOO) analysis revealed that removal of the *Leone* et al *(2001)* study produced the greatest reduction in the I^2^ heterogeneity from 90.95% to 76.75% compared to removal of other studies (supplementary Table 1). Subsequently, the *Leone* et al *(2001)* study was removed from the final meta-analysis. The remaining seven studies reported the proportion of family history in CH between 2 and 11%. The estimated true proportion of CH patients with a positive family history was 6.27% (95% CI:4.65–8.40%) and overall I^2^ of 73% (Fig. 2).

Moderator analysis was then performed in order to identify any potential confounding variables, in particular: the year of publication, sample size, and study design. Both sample size and year of publication did not show evidence of influencing the study outcome (*p* > 0.05).

**Fig. 3:**
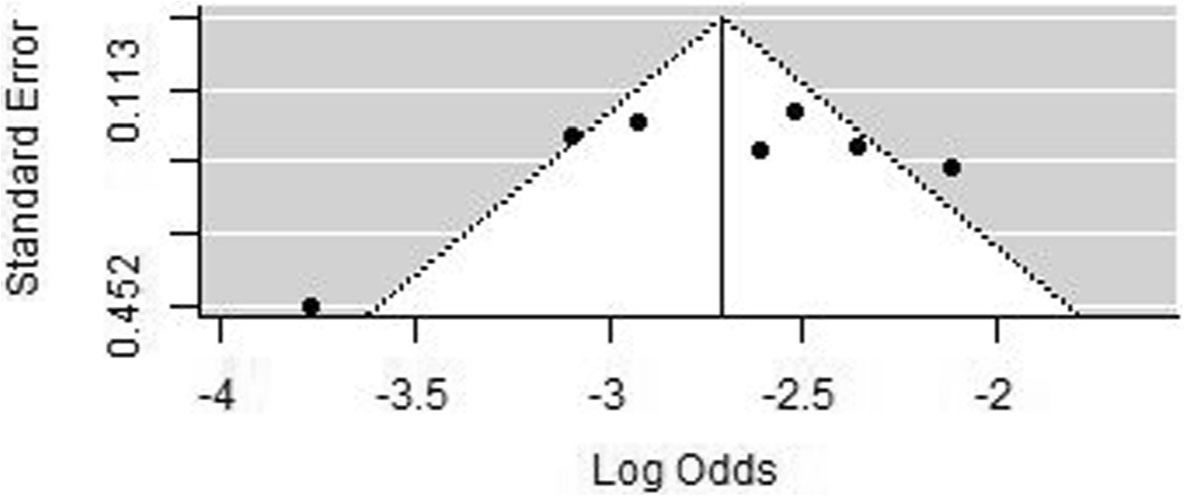
Funnel plot to assess potential publication bias. The x-axis shows the estimated prevalence (log odds) compared to the y axis which shows study precision in the seven selected studies

Finally, in the seven studies with outliers removed we assessed potential publication bias using a funnel plot and Egger regression testing. Funnel plots were roughly symmetrical (Fig. 3). Eggers test was not significant (*p* = 0.1701) indicating no clear evidence of publication bias. Performing the same analysis with the inclusion of the O’Connor (2020) cohort did not affect this result (*p* = 0.1127).

### Estimation of family history prevalence in male and female patients

**Fig. 4:**
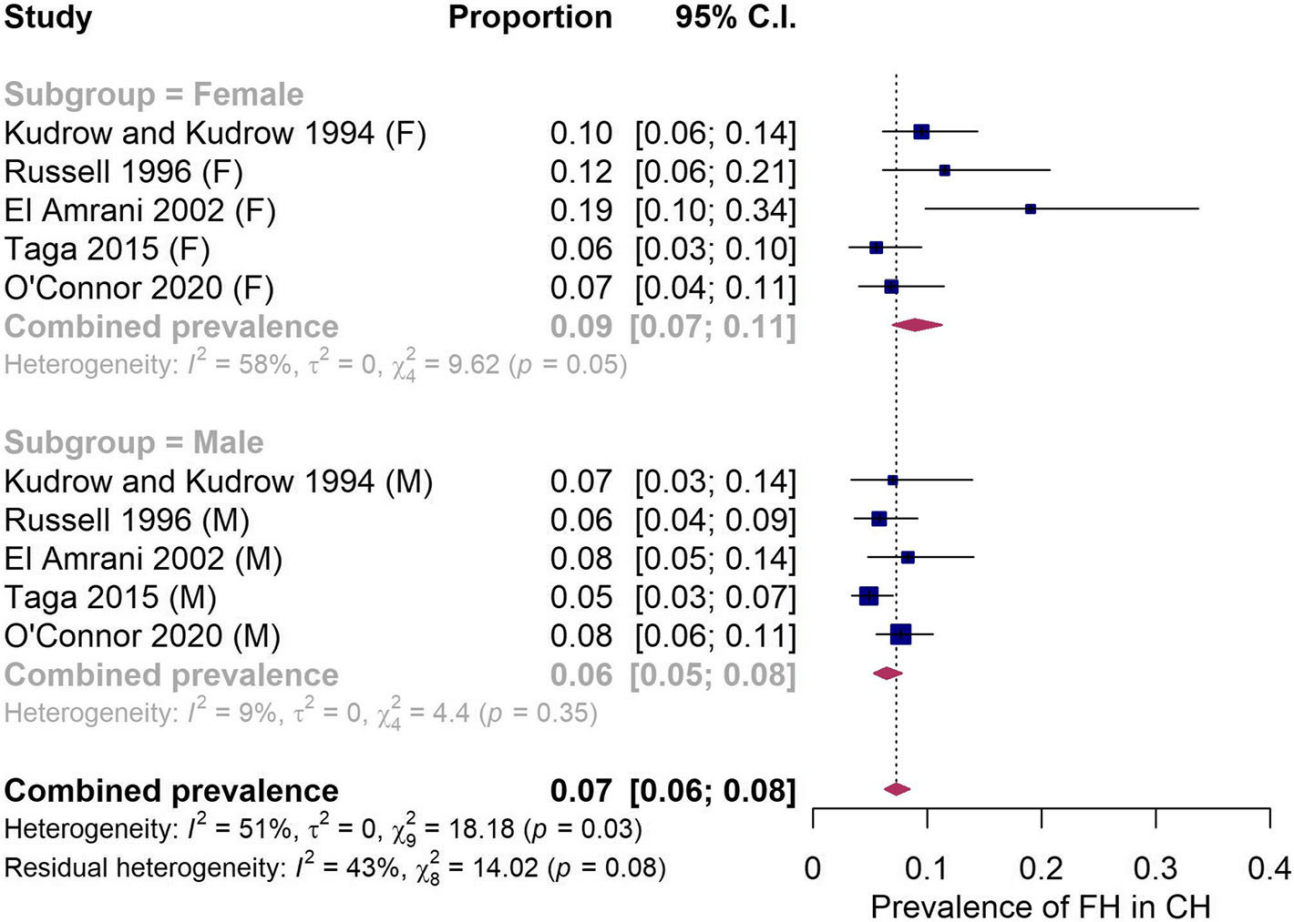
A random effects model was fitted to each subgroup which estimates the true prevalence of family history in female and male cluster headache patients. The study author and year (study), total number of cases with a positive family history (cases), total number of participants (total), prevalence proportion (prevalence) and 95% confidence intervals (95% C.I) are displayed along with measures of study heterogeneity. All values rounded to one significant figure

Despite the overall prevalence of CH being higher in males, a number of the identified studies reported an increased prevalence of family history of CH in females compared to males. We therefore conducted a separate analysis including only those studies which reported family history in males and females separately. Overall the fitted models for the subgroups estimated the prevalence of familial CH at 6.47% (95% CI: 5.27–7.92%) and 9.26% (95% CI: 6.29–13.43%) for males and females respectively (Fig. 4). The overall I^2^ for the male only model was just 9.14% and heterogeneity was no longer statistically significant (*p* = 0.354), while the I^2^ for the female model was 58.42% and remained significant (*p* = 0.047). While the summary estimate was larger for females than males, the results of the test of moderators revealed the subgroup summary estimates were not significant (*p* = 0.106). Therefore, we combined the estimates, producing a similar, albeit slightly higher estimate as our initial analysis of 6.98% (95% CI: 5.83–8.35).

**Fig. 5:**
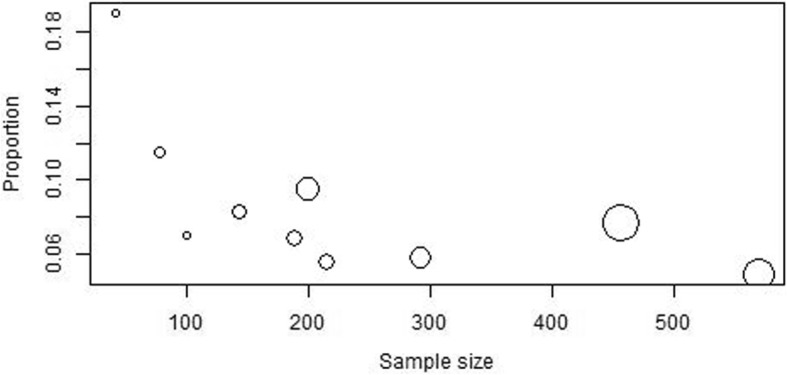
Funnel plot to assess potential publication bias. The x axis shows the estimated prevalence (log odds) compared to the y axis which shows study precision in the seven selected studies

Moderator analysis revealed an association between the model estimates and study size (*p* = 0.0176) (Fig. 5). The R^2^ indicated that approximately 64.2% of the true heterogeneity in the observed effect sizes are accounted for by sample size. This may potentially explain the heterogeneity seen in the female-only estimates as overall there were fewer females across studies The year of publication did not significantly influence the estimates (*p* = 0.2186).

**Fig. 6:**
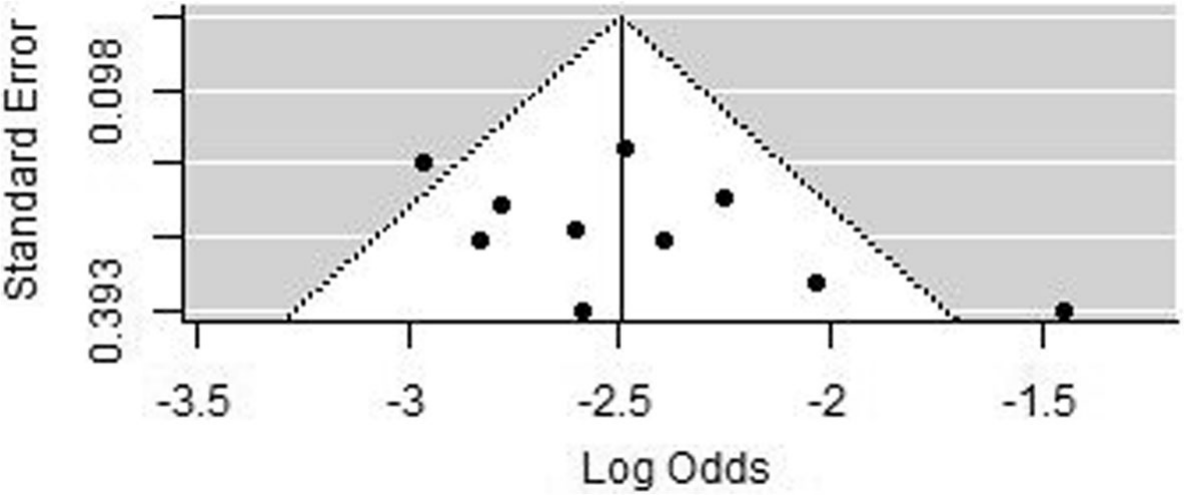
Funnel plot which is symmetrical showing no significant publication bias

Finally, as before, a funnel plot (Fig. 6) showed low evidence of asymmetry (*p* = 0.071). We, therefore, concluded that there was no significant publication bias within our analysis.

## Discussion

Previously, a number of studies have attempted to report the prevalence of family history in CH patients. Despite this, the exact prevalence of familial CH remains disputed, with some studies estimating a prevalence as low as 2.25% and others as high as 20% [13, 14]. Here, employing a robust systematic review and meta-analysis, we provide a true prevalence of familial CH of approximately 6.27%. To our knowledge, our analysis provides the most accurate estimation of familial CH to date.

Several epidemiology studies have reported higher prevalence of familial CH, possibly reflecting inflated estimations [14, 35, 36]. This disparity between studies is likely multifactorial. Notably, we excluded studies from our systematic review that lacked a confirmed clinical diagnosis in affected relatives. The high percentage of misdiagnosis or delay in diagnosis of CH by physicians is testament to the specialist clinical expertise required to provide an accurate diagnosis [37–39]. Our unpublished cohort was representative of this challenge, whereby a diagnosis of CH was inappropriately assigned to relatives by 18 (27.3%) probands. Therefore, clinical verification of a presumed diagnosis of CH in a relative should be a critical requirement in any study reporting family history.

The high degree of heterogeneity between studies included in our analysis is likely due to a number of factors including population stratification, differing reporting methods, an ambiguous definition of family history, variation in diagnostic criteria, and atypical phenotypes. Through ascertaining which studies adhered to strict eligibility criteria, we were able to homogenize data and derive a pooled estimate for the frequency of family history in patients with CH. Of note, the removal of the *Leone* et al *(2001)* study, as an outlier noticeably reduced the heterogeneity in our analysis. The considerably higher rate of family history in this cohort [20% (*n* = 44/220)] was attributed to the mode of data collection. Probands who were directly interviewed reported a considerably higher rate of family history compared with those recruited by postal questionnaire. Furthermore, a significant proportion of relatives were previously undiagnosed, investigated as part of the study and subsequently received a diagnosis. As this study was conducted almost twenty years ago, the under-diagnosis of CH is not unsurprising. However, this may also be compounded by intra-familial clinical variability and presence of phenotypes atypical for CH. The documented preponderance of relatives with atypical CH in these studies complicates this further [40, 41]. These cases are often omitted from epidemiological studies as they do not strictly fulfil diagnostic criterion, but perhaps represent part of a clinical spectrum associated with intergenerational genetic variation.

The high degree of variance in the reported estimates was illuminated further by a gender segregated analysis, which revealed that although the prevalence of family history was higher in females than in male probands, this difference was not significantly different. This conflicts with some reports which found a significant difference between gender (*Kudrow and Kudrow 1994, Taga 2015*). An explanation for this seemingly increased prevalence of family history in females is that CH is more common in males, therefore published studies tend to have larger numbers of male probands. Thus, the estimates of family history prevalence are more precise for males than females. We conducted a moderator analysis which revealed that study size influences the estimated prevalence. This potentially explains the discordance in observed prevalence between genders as the median number of male probands was 54% higher than the number of female probands across the five studies in our segregated analysis. Therefore, while we can estimate the prevalence of family history in male CH patients with a high degree of confidence, we found no convincing evidence that there is an increased prevalence of familial CH in females. Ultimately, further studies with a larger number of female probands are needed to determine any difference between genders, though this will likely have logistical challenges.

Finally, although lower than some previous estimates, our results nevertheless add to evidence suggesting a familial aggregation of CH and a role of genetic variation in its aetiology. This is further supported by several reports of concordance of CH reported amongst monozygotic twins [42–45], and genetic studies demonstrating association with variation in candidate genes [46–49]. Conceivably, one can hypothesize that families share similar environmental risk factors which can also contribute to the development of the CH phenotype. However, it is difficult to ignore consistent evidence showing CH to be more common in related individuals than in the general population, implying a possible genetic predisposition. Furthermore, an emphasis on familial history as part of clinical assessment could potentially lead to an earlier diagnosis and more efficient treatment of affected individuals.

The exact contribution of familial risk to CH is not yet clearly understood and is complicated by complex pedigrees which often demonstrate reduced penetrance [28]. Essentially, large sufficiently powered population-based studies are needed to ascertain an accurate estimation of genetic risk. This would further inform genetic studies and help provide optimal genetic counselling to sufferers and their families.

### Limitations

Our study is limited by its dependence on the interpretation of published data which limits our ability to explore clinical data in independent studies and provide a rigorous evaluation of factors influencing family history. We are also limited by potential recall bias whereby patients with CH are more likely to recount symptoms of the condition in a relative, than those without CH. Furthermore, restricting inclusion to studies where CH was confirmed in a relative, while improving accuracy, removed larger, population-based studies from our analysis. The small number of studies were also confined to high income settings in North America and Europe which impedes generalisability. Finally, despite an exhaustive search strategy across several databases with no restrictions, it is possible that relevant studies were erroneously omitted.

## Conclusion

In this systematic review and meta-analysis, we predict the prevalence of family history in CH to be approximately 6.27%. Additionally, contrary to previous findings, we were unable to confirm higher rates of familial history in female suffers. These results provide a robust estimation of the prevalence of familial CH and support the hypothesis of a potential genetic risk factors predisposing to the condition.

## Supplementary information

**Additional file 1: Supplementary Figure 1.** Density plot confirming normality following transformation of data.

**Additional file 2: Supplementary Figure 2.** Analysis involved random effects model which included all the identified studies estimating the prevalence of family history.

**Additional file 3: Supplementary Figure 3.** Diagnostic plots indicating the presence of an outlier in the estimation of relative proportion of effected probands with positive family history of CH.

**Additional file 4: Supplementary Table 1.** Leave one out (LOO) analysis shows that removal of Leone (2001) study reduced the I^2^ heterogeneity from 90.95% to 76.75%*.*

## Data Availability

Not Applicable.

## Abbreviations

CH: Chronic cluster headache
TAC: Trigemino-autonomic cephalalgia
PROSPERO: The International Prospective Register of Systematic Reviews
PRISMA- P: Preferred Reporting Items for Systematic Reviews and Meta-analysis Protocols
IHS: International Headache Society
ICHD3β: International Classification of Headache Disorders - third edition
LOO: Leave-One-Out analyses
DFFITS: Difference in fits values
