# Supplementary figures and images for: Prevalence of familial cluster headache: a systematic review and meta-analysis
Source: J Headache Pain. 2020 Apr 25;21(1):37. doi: 10.1186/s10194-020-01101-w (PMC7183702; doi:10.1186/s10194-020-01101-w)

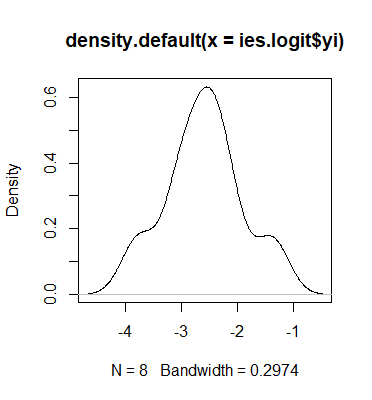

Supplement: Supplementary file 1 — Additional file 1: Supplementary Figure 1. Density plot confirming normality following transformation of data. [file 10194_2020_1101_MOESM1_ESM.png]

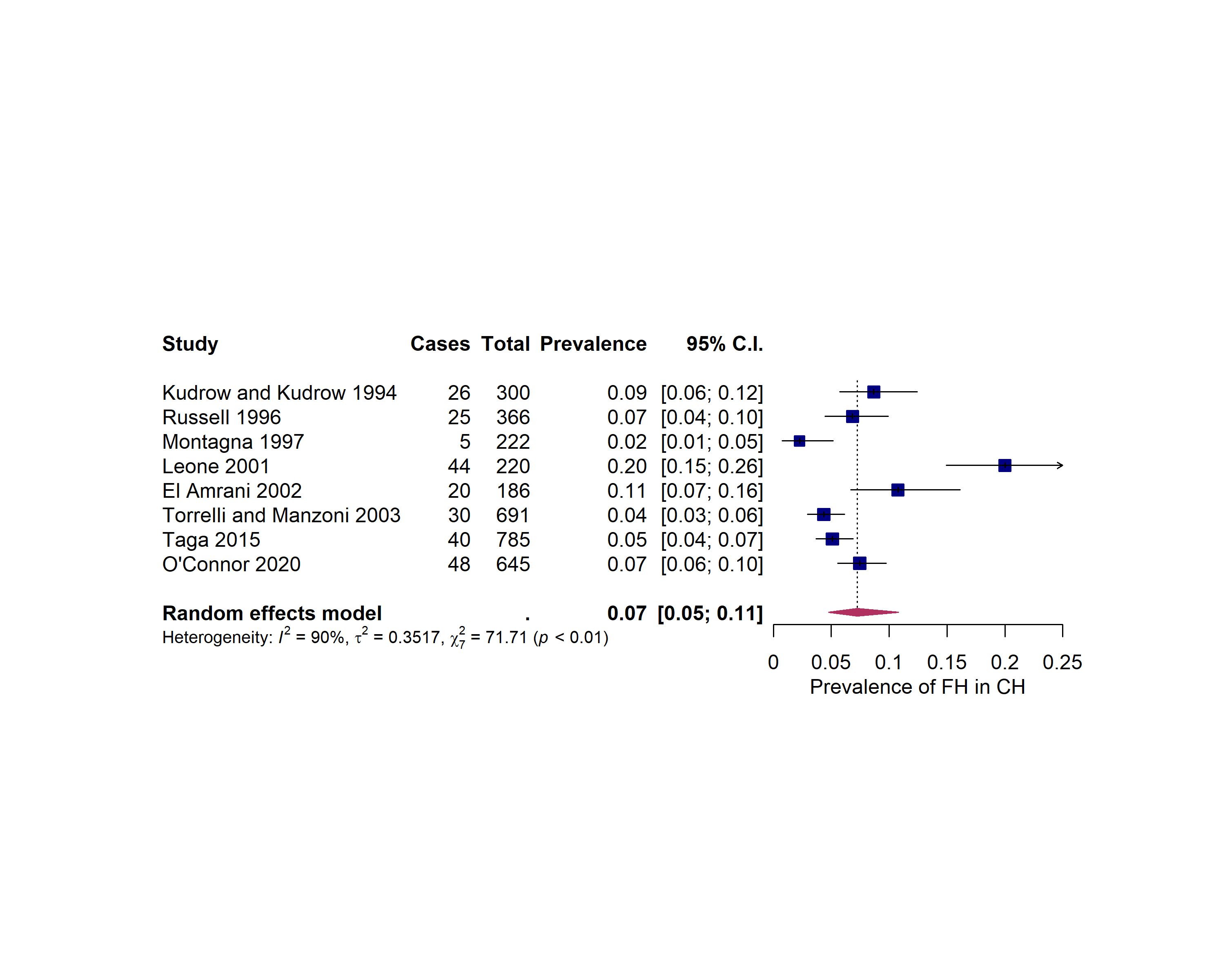

Supplement: Supplementary file 2 — Additional file 2: Supplementary Figure 2. Analysis involved random effects model which included all the identified studies estimating the prevalence of family history. [file 10194_2020_1101_MOESM2_ESM.jpg]

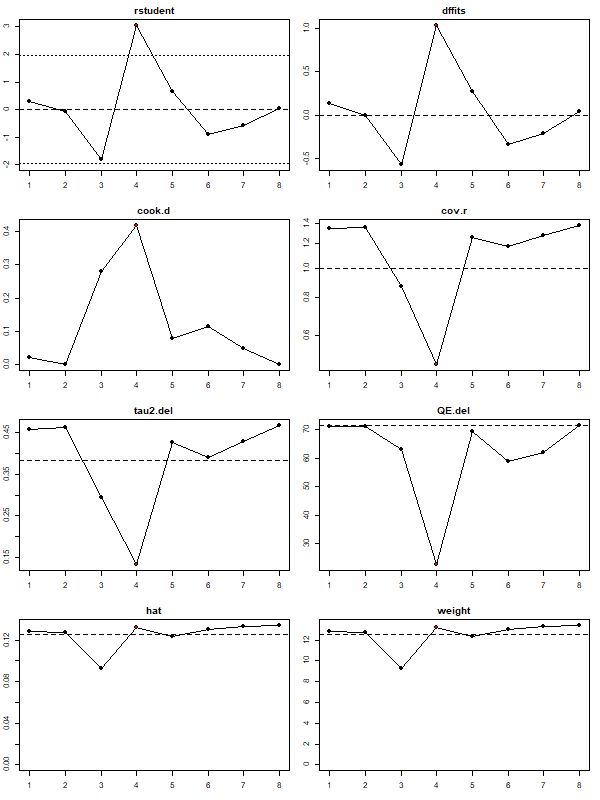

Supplement: Supplementary file 3 — Additional file 3: Supplementary Figure 3. Diagnostic plots indicating the presence of an outlier in the estimation of relative proportion of effected probands with positive family history of CH. [file 10194_2020_1101_MOESM3_ESM.jpg]
