# Supplementary material for: Prevalence of familial cluster headache: a systematic review and meta-analysis
Source: J Headache Pain. 2020 Apr 25;21(1):37. doi: 10.1186/s10194-020-01101-w (PMC7183702; doi:10.1186/s10194-020-01101-w)
Supplement: Supplementary file 4 — Additional file 4: Supplementary Table 1. Leave one out (LOO) analysis shows that removal of Leone (2001) study reduced the I2 heterogeneity from 90.95% to 76.75%. [file 10194_2020_1101_MOESM4_ESM.docx]

**Supplementary table 1:** Leave one out (LOO) analysis shows that removal of Leone (2001) study reduced the I^2^ heterogeneity from 90.95% to 76.75%*.*

| **Study** | **estimate** | **zval** | **pval** | **ci.lb** | **ci.ub** | **Q** | **Qp** | **tau2** | **I2** | **H2** |
| --- | --- | --- | --- | --- | --- | --- | --- | --- | --- | --- |
| **Kudrow and Kudrow 1994** | 0.068522 | -8.03122 | 9.65E-16 | 0.037454 | 0.122093 | 71.2786 | 5.55E-14 | 0.573291 | 93.12907 | 14.55407 |
| **Russell 1996** | 0.071372 | -7.8481 | 4.22E-15 | 0.038919 | 0.127302 | 70.89675 | 6.67E-14 | 0.5812 | 93.23762 | 14.78768 |
| **Montagna 1997** | 0.081983 | -9.40515 | 5.20E-21 | 0.051217 | 0.128724 | 62.86965 | 3.10E-12 | 0.357634 | 90.75166 | 10.81275 |
| **Leone 2001** | 0.060042 | -13.8083 | 2.27E-43 | 0.041438 | 0.086248 | 20.87984 | 0.000854 | 0.181164 | 76.74742 | 4.957224 |
| **El Amrani 2002** | 0.066217 | -8.41919 | 3.79E-17 | 0.036885 | 0.116062 | 69.25927 | 1.46E-13 | 0.535263 | 92.97622 | 14.23735 |
| **Torrelli and Manzoni 2003** | 0.0775 | -8.10609 | 5.23E-16 | 0.044122 | 0.132626 | 57.60195 | 3.80E-11 | 0.499239 | 91.92747 | 12.3877 |
| **Taga 2015** | 0.075286 | -7.88475 | 3.15E-15 | 0.04182 | 0.131849 | 60.86631 | 8.05E-12 | 0.544286 | 92.12084 | 12.69171 |
